# Large volume syringe pump extruder for desktop 3D printers

**DOI:** 10.1016/j.ohx.2018.02.001

**Published:** 2018-02-12

**Authors:** Kira Pusch, Thomas J. Hinton, Adam W. Feinberg

**Affiliations:** a Department of Materials Science & Engineering, Carnegie Mellon University, United States; b Department of Biomedical Engineering, Carnegie Mellon University, United States

**Keywords:** Additive manufacturing, 3D bioprinting, Embedded printing, FRESH, Soft materials extrusion

## Abstract

Syringe pump extruders are required for a wide range of 3D printing applications, including bioprinting, embedded printing, and food printing. However, the mass of the syringe becomes a major challenge for most printing platforms, requiring compromises in speed, resolution and/or volume. To address these issues, we have designed a syringe pump large volume extruder (LVE) that is compatible with low-cost, open source 3D printers, and herein demonstrate its performance on a PrintrBot Simple Metal. Key aspects of the LVE include: (1) it is open source and compatible with open source hardware and software, making it inexpensive and widely accessible to the 3D printing community, (2) it utilizes a standard 60 mL syringe as its ink reservoir, effectively increasing print volume of the average bioprinter, (3) it is capable of retraction and high speed movements, and (4) it can print fluids using nozzle diameters as small as 100 µm, enabling the printing of complex shapes/objects when used in conjunction with the freeform reversible embedding of suspended hydrogels (FRESH) 3D printing method. Printing performance of the LVE is demonstrated by utilizing alginate as a model biomaterial ink to fabricate parametric CAD models and standard calibration objects.

## 1. Hardware in context

### 1.1. Introduction

Syringe-pump-based 3D printers can be generically broken down into two categories: bioprinters and paste/clay extruders. Bioprinters are designed for high-precision printing of biopolymers and cells, and usually feature low-volume syringes and small-diameter nozzles (< 250 µm) to print small objects [1–3]. Conversely, paste extruders are designed for high-capacity, often featuring large material reservoirs and large-diameter nozzles (up to 4 mm). The broad category of pastes encompasses materials that have shear thinning and/or yield-stress rheological behavior and includes clays, ceramics, composite resins, foods like frosting, cements, and materials of similar consistencies. Paste extruders are subject to an array of performance issues, often printing at slow speeds and producing simple geometries. Further, most syringe-pump-based printers have issues with retraction, which is the ability of a 3D printer to stop and reverse extrusion prior to movement between printed portions of each layer. Retraction is important because it prevents material from leaking or oozing during non-extruding moves, thereby preventing unintentional extrusions or thin strings of material that can reduce print fidelity.

**Fig. 1:**
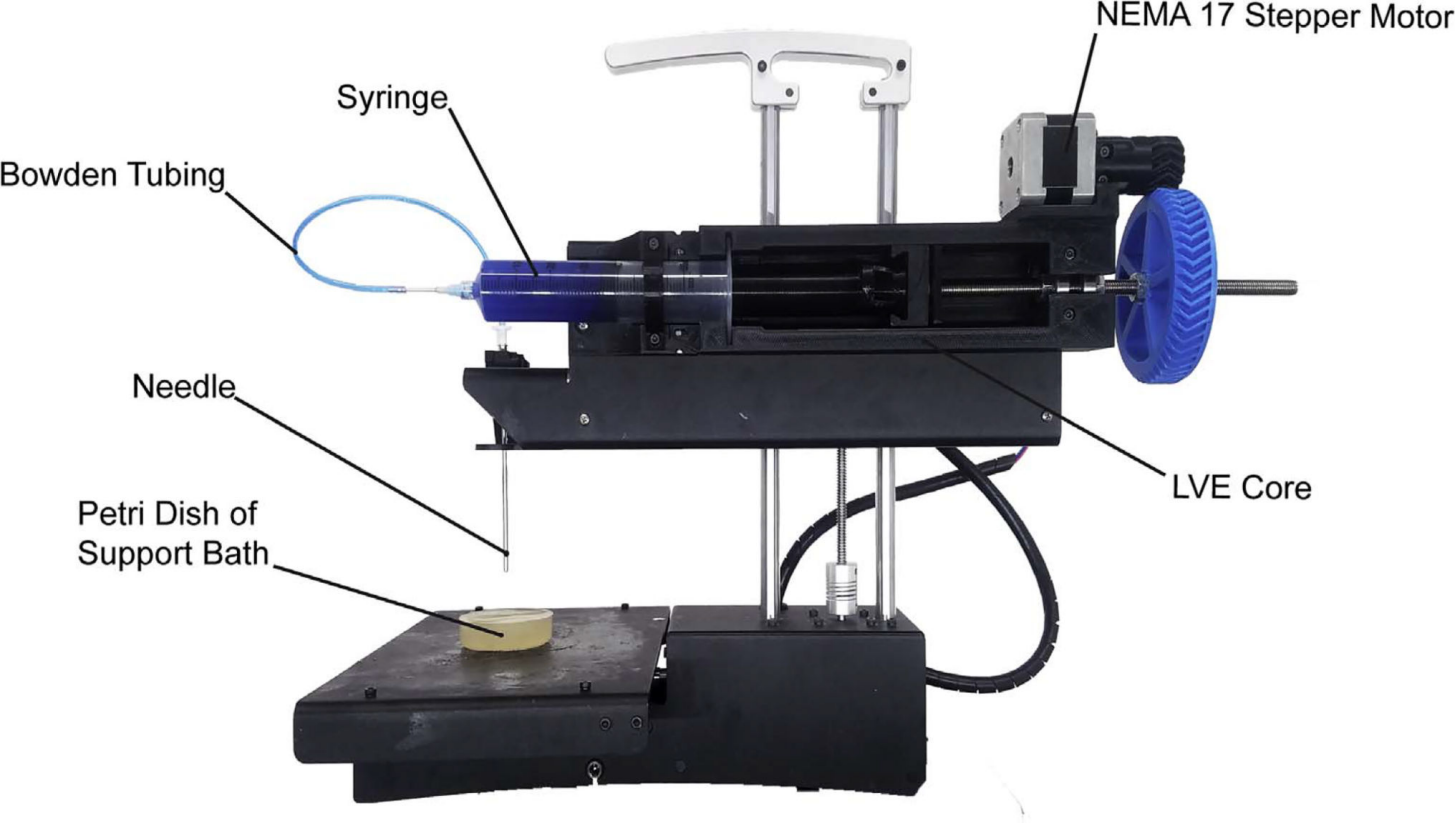
PrintrBot Simple Metal modified with the LVE for FRESH printing. The build plate of the printer holds a Petri dish of FRESH support bath into which the needle is lowered to perform printing. The Bowden tubing, comprised of polyurethane and acquired online from Grainger (See *Bill of Materials*), separates the 60 mL disposable syringe (here containing Alcian-Blue-dyed alginate) from the needle, allowing the 3D printed LVE core to be mounted to the frame of the PrintrBot as shown. The LVE leadscrew is also driven by the stock NEMA 17 stepper motor that came with the PrintrBot. The stepper motor has simply been removed from the stock hot end assembly and screwed into the LVE assembly as shown; no other alterations to the motor are required for this set up.

The Large Volume Extruder (LVE) was developed to print a range of materials, including biopolymers, hydrogels, pastes, epoxies, and other shear thinning and yield-stress fluids (Fig. 1). Specifically, goals of the LVE were to: (1) minimize cost and difficulty of fabrication by utilizing standard syringe sizes and luer-fittings, (2) increase bioprinter ink volume, and (3) increase performance of paste extruders by enabling use of small nozzle sizes and increased speeds. The result is an inexpensive, 3D-printable, 60 mL-volume, open source syringe pump extruder that can retract, operate at high speeds, and fabricate complex objects via the freeform reversible embedding of suspended hydrogels (FRESH) 3D bioprinting method [4].

### 1.2. Cost

Commercial 3D bioprinters range in cost from $10,000 to > $200,000 and are typically proprietary machines, closed source, and difficult to modify. All these issues serve as a barrier to innovation in research. To lower the cost of entry, moderately-priced large volume paste extruders such as the Discov3ry extruder (https://www.structur3d.io/), which has a starting price of $1300, have become available for purchase. While these are more cost-accessible than commercial 3D bioprinters, such drop-in modifications are not specifically designed for retraction and are typically used with large-diameter nozzles. Some paste extrusion systems are also pneumatically-driven, requiring separate and bulky air pressure supply units that add to the overall cost of the system.

There are currently open source projects and companies that offer affordable syringe pump extruder modifications for use with low-cost 3D printers. For example, the PrintrBot Food & Paste Extruder (https://printrbot.com/shop/paste-food-extruder/) is low cost, however, this was designed for use with the PrintrBot Simple Metal, requires rewiring of the printer during installation, and is not easily translated to other systems. It is important to note that several open source designs, such as the “Universal Paste Extruder” by RichRap (https://www.thingiverse.com/thing:20733) have become available online for retrofitting consumer 3D thermoplastic printers; however, these designs are often limited by either small build volume or the inability to retract during printing [5–8]. The Replistruder by TJ Hinton (https://www.youmagine.com/designs/replistruder-v2-5-syringe-pump-extruder) is also an open source, custom-designed syringe-based extruder, specifically designed for precision hydrogel deposition via the FRESH method. The Replistruder has been used to demonstrate the 3D printing of mechanically robust, biomimetic structures with high repeatability using collagen, fibrin, and Matrigel, however it too is limited in volume and utilizes, at maximum, a 10 mL syringe as its ink reservoir [4].

To address the issue of cost and to make large volume fluid extrusion accessible to the average desktop printer user, we designed the LVE for use with most open source desktop printers and software. The total cost of the LVE is only $49.27 (see *Bill of Materials*), and is reduced significantly by the fact that it can mostly be 3D printed. A desktop thermoplastic 3D printer can therefore be used to modify itself by printing the LVE and installing it on the same machine. The other components, including the Bowden tubing, bearings, stopcock, needles, and threaded rod, are inexpensive and easy to acquire. Except for the threaded rod, bearings, and nuts and bolts, all these parts are also disposable and readily replaced as needed. The LVE is compatible with standard 60 mL syringes, per ISO 7886-2:1996 [9], and luer-lock needles, both of which are widely available online.

### 1.3. Volume

Current syringe pump extruders are typically large volume or high resolution, but not both. Commercial and open source syringe-based bioprinters are often direct-extrusion systems where the fluid reservoir is incorporated into the extruder carriage. This is of central importance for large volume syringes, where the added mass to the carriage increases vibration and unwanted motion artifacts during printing. Accordingly, bioprinters generally utilize 10 mL syringes to minimize extruder carriage payload, but this leaves the drawback of limited volume capacity. The syringe pump extruder developed by Hinton et al. for use with the FRESH 3D printing method, termed the Replistruder, clearly illustrates the tradeoff between performance and volume in bioprinters. The Replistruder was designed to provide retraction, and the ability to accurately stop and reverse extrusion enabled the bioprinting of highly complex and biomimetic constructs using an alginate bioink [4]. As previously stated, the Replistruder system is limited in its ability to print large parts because at maximum it uses a 10 mL syringe as the reservoir of printing material. While the Replistruder could be modified to increase the syringe volume, the added mass on the carriage would negatively impact printing performance. As another example, Lewis and co-workers developed a highly accurate pneumatic-driven syringe extruder, which while not exclusively a bioprinter, achieved high-fidelity epoxy and polymer printing with a low-volume material reservoir [10]. In contrast, commercial paste extruders such as the Fab@Home Scientist Printer (http://www.scientist3d.com/), PrintrBot Paste & Extruder (https://printrbot.com/shop/paste-food-extruder/), Discov3ry Paste Extruder (https://www.structur3d.io/discov3ry-2-complete/), and ZMorph Thick Paste Extruder (https://zmorph3d.com/products/toolheads/thick-paste-extruder) feature 30 to 100 mL syringes as their material reservoirs, but what they gain in volume, they lose in performance and precision. These larger material reservoirs typically use large diameter nozzles that reduce resolution and print at low speeds to compensate for their more massive payloads [5,6,11–13]. Some large volume systems utilize a Bowden approach to minimize weight on the extruder assembly by separating the material reservoir from the nozzle via tubing, obviating the need for dramatic speed reduction. Such systems are typically pneumatically driven, but this has limitations, including extrusion pressure that varies with the amount of material remaining in the reservoir and the need to apply vacuum during printing to enable retraction. Pneumatically driven systems also feature signal-response delays that are dependent on both the rheological properties (e.g., Newtonian, shear thinning, thixotropic) of the material being extruded and the volume of the material within the reservoir. The LVE was designed to use a stepper motor to achieve retraction through straightforward reversal of direction, and to apply consistent pressure using a standard 60 mL syringe.

### 1.4. Printer performance

A vast majority of syringe extruders operate without the ability to retract and are therefore susceptible to material leakage during non-print moves. This can result in low print quality and artifacts such as fine, hair-like strands of unwanted material, bulging corners, and over-extrusion. For example, the EnvisionTEC Bioplotter (https://envisiontec.com/3d-printers/3d-bioplotter/), which has been used to print both biopolymers and pastes, is pneumatically driven and compensates for its inability to retract by including an automated nozzle cleaning station used between each layer during printing. This process involves pausing during printing to purge pressure build up in the system, wiping excess material leaking from the extruder, and cleaning the nozzle against a set of bristles. The result is a significant increase in the print time of even small objects. Conversely, the LVE is capable of retraction and is therefore able to print highly complex objects without significant oozing of the print material. Also, leadscrew driven extruders such as the LVE deposit consistent amounts of material no matter how full the syringe is or how viscous the material is, making them more precise than other methods of extrusion.

Commercial paste extruders run at slow speeds, for example the PrintrBot Paste & Food Extruder, Fab@Home Scientist Printer, Discov3ry, and ZMorph Thick Paste Extruder all recommend speeds ≤ 20 mm/s [5,6]. Bioprinters, which utilize smaller volumes of material, are capable of comparatively faster speeds while extruding much smaller volumes of fluid This is particularly true of many commercial bioprinters that are equipped with expensive, high quality motions systems. For example, the EnvisionTEC Bioplotter recommends print speeds less than 40.5 mm/s. However, this still falls short of current desktop printers that can achieve 300 mm/s movements using a Bowden style setup [14,15].

The LVE adds minimal payload to a given printer’s movements, resting its weight on the printer frame and not the extruder carriage. This setup maximizes print speed by removing most of the extruder mass from the moving nozzle assembly, and it minimizes vibration of the printer during printing. Specifically, excessive mass on the extruder carriage, especially during high speed acceleration and deceleration, can cause vibration of light weight 3D printers and skipping of the stepper motors, which can cause printing defects such as a “layer-shift” artifact that affects all subsequent layers with an offset in the direction of the skip. The LVE is capable of operating at the maximum speed of the printer we are using, and with our design have had no issues with printer stability or motor skipping. In the case of a PrintrBot Simple Metal, this is approximately 80 mm/s. Additionally, the LVE is, in principle, compatible with all open source printers. It was designed for use with the NEMA 17 stepper motors found on most open source 3D printers, enabling installation with minimal rewiring or removal of parts. Use of Bowden-style extrusion means that the LVE can be mounted to the printer frame and the default thermoplastic hot-end extruder can be swapped out for a needle connected to the LVE via rigid tubing. Furthermore, the LVE is compatible with a range of needle sizes, unlike paste extruders that often use large diameter nozzles up to 4 mm. Here we demonstrate that a stainless steel blunt tip needle (250 µm inner diameter) can be used to produce prints with 120 µm layer height. Accordingly, the LVE is not subject to the tradeoff between print volume and print detail that commonly limits other syringe extruders.

## 2. Hardware description

**Fig. 2:**
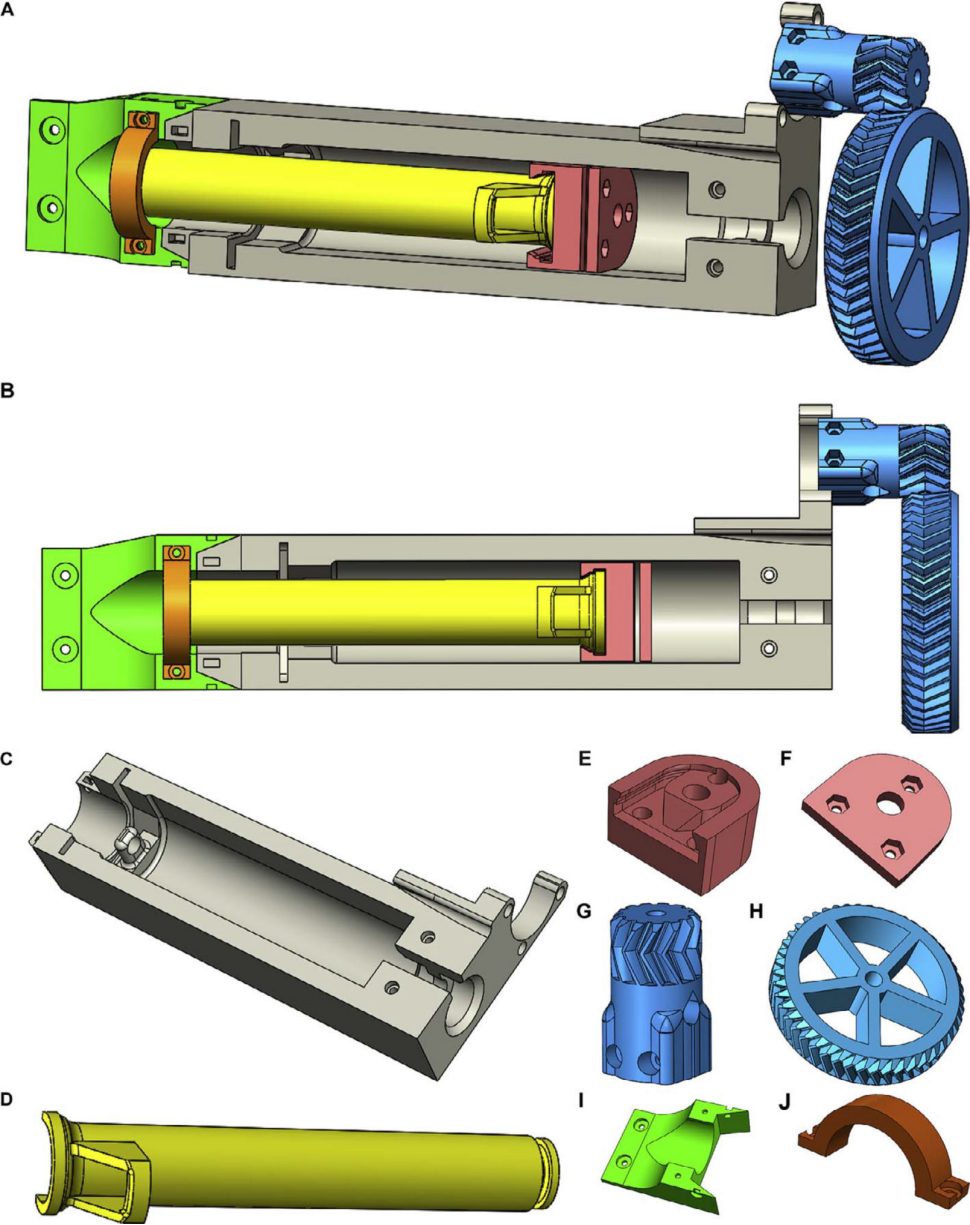
3D CAD models of all 3D printed LVE components. (A) Angled view of color-coded assembly. (B) Front view of color-coded assembly. Angled views of 3D printed LVE components consisting of (C) LVE core, (D) Syringe Plunger, (E) Nut Shuttle, (F) Nut Shuttle Plate, (G) Motor-Mounted Gear, (H) Threaded Rod-Mounted Gear, (I) PrintrBot-Specific LVE Adapter, and (J) Syringe Collar.

**Fig. 3:**
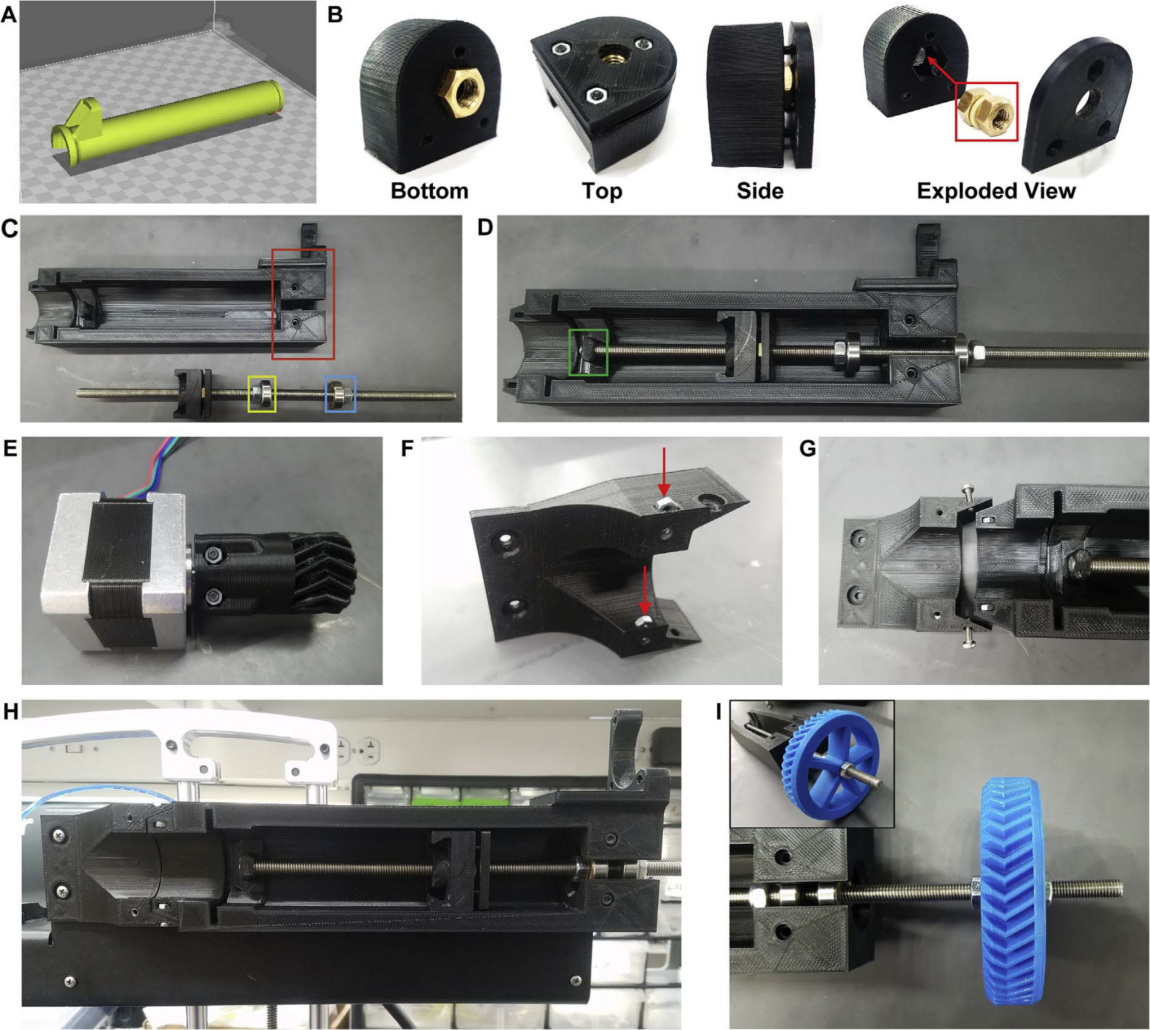
Assembly of Printed Parts. (A) Screenshot of the Syringe Plunger in slicing software (Cura) in the orientation recommended for printing. The Syringe Plunger requires support structures during printing. (B) Nut Shuttle assembly; the brass nuts are sandwiched and put under compression by the nut shuttle plate which is screwed into place using three M3 × 10 mm hex bolts and three M3 hex nuts. (C) LVE Core with red box outlining the bearing housing referenced in step five of the build instructions. The yellow box encircles the nut and bearing spaced along the leadscrew to the left of the bearing housing. The blue box encircles the nut and bearing spaced along the leadscrew to the right of the bearing housing (which can be screwed on after the threaded rod is installed in the LVE Core). (D) LVE core with the rod inserted into place following proper spacing of hardware components along the threaded rod. The green box encircles the guide post in which the threaded rod is sitting. Note that the rod does not sit past the guide post. (E) NEMA 17 stepper motor with small gear attached. (F) Syringe Guide with red arrows indicating the slots into which M3 nuts should be inserted to enable the syringe collar to be screwed into place after loading the syringe. (G) Top-down view of Syringe Guide and LVE Core. (H) LVE system mounted on a PrintrBot Simple Metal. (I) Large Gear affixed to threaded rod with counter-tightened M8 steel nuts.

The LVE combines effective design elements from existing extrusion systems into an easily printed, assembled, and installed device. Specifically, it is lightweight, leadscrew driven, capable of retraction, inexpensive, open source, and designed for ease of fabrication, installation, and operation by the average user. Many of the key components of the LVE system are 3D printed, making it inexpensive but also easily modified as needed. Designs are licensed as open source under a CC BY-SA 4.0 license. Non-printed parts include standard hardware such as bearings, M3 and M8 nuts and bolts, an M8 threaded steel rod, luer-fitting needles, polyurethane tubing, and several luer-fitting adapters. The LVE consists of four main parts: (1) the core, which houses the motor, (2) the geared leadscrew transmission which actuates the plunger of the syringe, (3) the plunger adapter or “nut shuttle,” which fixes the plunger to the leadscrew and enables backwards movement of the plunger, and (4) the Bowden portion of the LVE which utilizes stiff, polyurethane tubing and a stainless steel 100 mm-long needle to separate the 250 µm needle from the syringe and allows it to sit further down in a container, such as a beaker, used during FRESH printing. Individual components are shown in CAD in Fig. 2, and printed versions are shown in Figs. 1 and 3.

### 2.1. LVE core

**Fig. 4:**
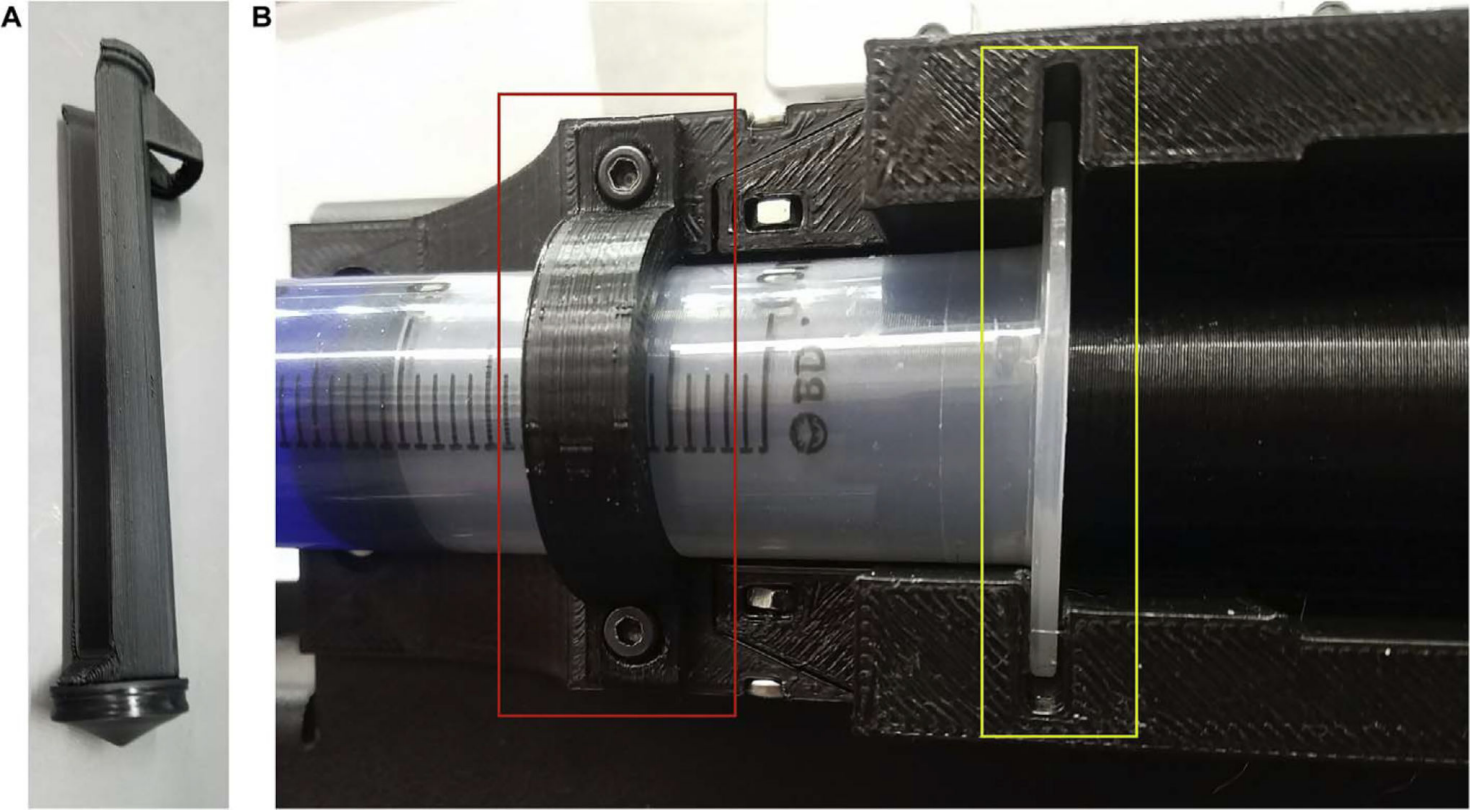
Syringe Preparation and Loading in LVE. (A) Image of 3D printed PLA Syringe Plunger with neoprene plunger tip installed. (B) Image of the LVE with syringe loaded; the red box designates the Syringe Collar which holds the syringe in place, and the yellow box outlines the slot in which the syringe flange sits.

The core of the LVE was designed to hold a disposable 60 mL BD syringe and is sized accordingly. The slot into which the syringe flange fits is designed specifically for compatibility with BD’s syringes (Fig. 4). The core bears most of the compressive load across the leadscrew during actuation; therefore, to maximize the stiffness and minimize bending in the system, the core was printed on a Lulzbot Taz6 with PLA filament and 100% rectilinear infill, which was found to produce a stiffer part. Tall side walls guide the 100% infill PLA syringe plunger along the axis of the leadscrew during printing, increasing the overall stiffness of the system. The LVE core can be 3D printed on its flat face and requires no supports during printing. Further, the core requires no post-processing (save for minor assembly steps such as snapping bearings into place) and need only be attached to the frame of the printer. During installation of the core on the PrintrBot Simple Metal, the thermoplastic extruder nozzle was removed from the PrintrBot and set aside, only being disassembled to the extent that the extruder stepper motor could be relocated (though remain plugged into the motherboard) to the designated spot on the LVE. Assembly is straightforward and translatable to other printing systems with accessible extruder motors and closely resembles the process described above.

### 2.2. Geared leadscrew transmission

The gear system utilizes two herringbone gears; a small, self-centering gear designed to fit onto the shaft of a NEMA 17 stepper motor meshes to a large gear fixed to the leadscrew, which controls the motion of the syringe plunger. The “steps-per-millimeter” of the printer’s extruder was changed to 9255.38 to accommodate for this modification, and this value represents the number of 1/16th microsteps required to advance the 60 mL plunger by 1 mm in either direction.

### 2.3. Nut shuttle actuator

Retraction in the LVE system is enabled through two nuts on the threaded leadscrew that are kept in compression and thus are free of mechanical slack. These nuts are compressed by a “nut shuttle plate,” which is screwed into the “nut shuttle” housing the nuts, effectively sandwiching the nuts between the plate and the plunger. Trapped in the nut shuttle assembly, the nuts serve to actuate the plunger of the syringe and enable precision movement of the plunger during printing. When the printer extrudes, the NEMA 17 stepper motor spins its shaft and the small gear clockwise. This motion causes the large gear and leadscrew to spin counterclockwise. The nuts sandwiched between the nut shuttle and the nut shuttle plate progress downward along the leadscrew, exerting pressure on the syringe plunger. Because the movement of the nuts are controlled by the leadscrew, and because the nuts are effectively fastened to the top of the plunger via the nut shuttle plate, the NEMA 17 stepper motor can retract by spinning counter-clockwise, resulting in a tensile, upward force on the syringe plunger.

### 2.4. Bowden components

**Fig. 5:**
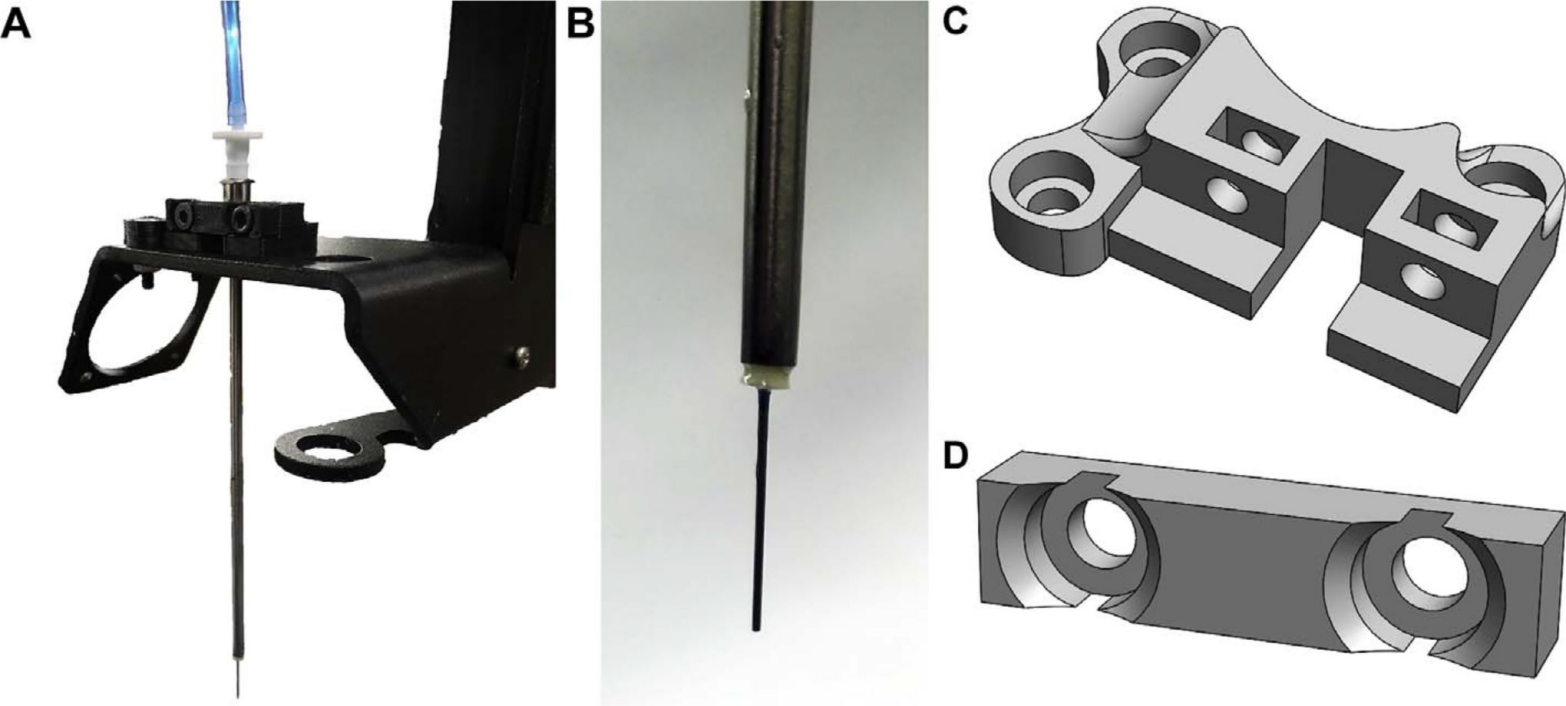
Epoxied Needles and PrintrBot-Specific Needle Adapter (A) Image of epoxied needles attached to PrintrBot via Needle Adapter. (B) Close-up image of epoxied needles. The epoxy, here a beige color, can clearly be seen between the 250 µm needle and the 100 mm, 18-gauge needle. (C) CAD model of PrintrBot-specific needle adapter in Solidworks. (D) Needle Collar; screws into the Needle Adapter using two M3 × 8 mm screws and two M3 nuts.

The LVE is a Bowden-style system, which is key to its functionality as a precise, high-volume fluid extruder. Bowden-style extruders allow for minimization of payload of the nozzle assembly, increasing performance and speed capabilities during 3D printer movements. On the PrintrBot Simple Metal, the payload is not separate from the XY gantry of the printer because the PrintrBot has no frame and therefore no external location on which to mount the LVE. Instead, the LVE is mounted in a horizontal position along the length of the Y-axis assembly, where the added mass can be evenly distributed. To perform FRESH printing inside large containers, an extension of the 250 µm needle was achieved by removing its luer fitting, sliding it into a 100 mm-long, 18-gauge dispensing needle, and epoxying it in place (Fig. 5A and B). Without needle extension, the printer is limited to printing objects that are as tall as standard dispensing needles, which are generally no longer than 25 mm. With the 250 µm needle extended as described, objects as tall as 75 mm can be fabricated. The Bowden tubing (see *Bill of Materials*) is polyurethane that can withstand up to 240 psi. Several components of the Bowden portion of the LVE needed to be purchased, including a 17-gauge needle to connect the syringe to the tubing, and a male-to-male luer adapter to connect the tubing to the 18-gauge needle. Further, as this system was implemented on a PrintrBot Simple Metal, an adapter to connect the dispensing needle to the PrintrBot frame was also designed (Fig. 5C and D). Connecting the needle to the extruder to other 3D printers is simply a matter of affixing the 100 mm-long needle to the extruder carriage.

### 2.5. Key aspects of the hardware

The LVE is useful to researchers that need to 3D print biological materials, pastes and similar fluid materials. It provides a number of design elements that provide advantages over existing open source and commercial programmable syringe extruders.

- The LVE hardware is designed to fit on an open source desktop printer, and can be easily adapted to fit on wide range of similar low-cost 3D printers
- Placement of the LVE on the printer minimizes the length of tubing connecting the syringe to the extrusion needle, reducing compliance in the system
- Using the NEMA 17 stepper motor to drive the syringe extruder means the LVE can be operated without additional hardware or software modifications
- The LVE is designed for rapid changes from extrusion to retraction, which is required of 3D printing fluids, in contrast to most programmable syringe pumps that are typically built for precision extrusion only
- It is straightforward to install and use
- The system is compatible with the published FRESH method of printing hydrogels [4]
- The system is similar in operation to standard FFF printing, making it accessible to users with desktop 3D printers
- The LVE can be used to print a wide range of materials including epoxies, fluid gels like collagen and alginate, and pastes

## 3. Design files

Design Files Summary

**Table:** 

Design file name	File type	Open source license	Location of the file
LVE Core(Fig. 2C)	CAD file & STL file	CC-BY-SA 4.0	https://3dprint.nih.gov/discover/3dpx-008366
Syringe Plunger(Fig. 2D)	CAD file & STL file	CC-BY-SA 4.0	https://3dprint.nih.gov/discover/3dpx-008366
Nut Shuttle(Fig. 2E)	CAD file & STL file	CC-BY-SA 4.0	https://3dprint.nih.gov/discover/3dpx-008366
Nut Shuttle Plate(Fig. 2F)	CAD file & STL file	CC-BY-SA 4.0	https://3dprint.nih.gov/discover/3dpx-008366
Small Gear(Fig. 2G)	CAD file & STL file	CC-BY-SA 4.0	https://3dprint.nih.gov/discover/3dpx-008366
Large Gear(Fig. 2H)	CAD file & STL file	CC-BY-SA 4.0	https://3dprint.nih.gov/discover/3dpx-008366
Syringe Guide(Fig. 2I)	CAD file & STL file	CC-BY-SA 4.0	https://3dprint.nih.gov/discover/3dpx-008366
Syringe Collar(Fig. 2J)	CAD file & STL file	CC-BY-SA 4.0	https://3dprint.nih.gov/discover/3dpx-008366
Needle Adapter(Fig. 4C)	CAD file & STL file	CC-BY-SA 4.0	https://3dprint.nih.gov/discover/3dpx-008366
Needle Collar(Fig. 4D)	CAD file & STL file	CC-BY-SA 4.0	https://3dprint.nih.gov/discover/3dpx-008366
250 µm Needle Jig(Fig. 7)	CAD file & STL file	CC-BY-SA 4.0	https://3dprint.nih.gov/discover/3dpx-008366
18-Gauge Needle Jig_short(Fig. 7)	CAD file & STL file	CC-BY-SA 4.0	https://3dprint.nih.gov/discover/3dpx-008366
18-Gauge Needle Jig_extended(Fig. 7)	CAD file & STL file	CC-BY-SA 4.0	https://3dprint.nih.gov/discover/3dpx-008366

**LVE Core (**Fig. 2C**):** The Core is the main component of the LVE and houses the drivetrain. The NEMA 17 stepper motor also mounts to the Core, and the leadscrew is centered in the LVE core by the guide post, which can be seen in Fig. 3D. Likewise, the slot to the left of the guide post is where the disposable 60 mL syringe snaps into place (Fig. 4B).
**Syringe Plunger (**Fig. 2D**):** Syringe plunger for replacement of stock plastic plunger that comes with the 60 mL BD syringe. The neoprene tip of the stock plunger must be removed and put onto this 3D printed version, which was designed to avoid collision with the guide post of the LVE core (Fig. 4A).
**Nut Shuttle (**Fig. 2E**) and Nut Shuttle Compression Plate (2F):** These pieces house the brass nuts responsible for enabling actuation of the syringe plunger – extrusion and retraction. The Nut Shuttle Plate (Fig. 2F) screws into the Nut Shuttle (Fig. 2E) to keep the brass nuts under compression and to minimize play in the system during printing. They can be screwed together with M3 hex nuts and M3 × 10 mm screws (see Fig. 3B).
**Small Gear (**Fig. 2G**):** The small gear mounts onto the NEMA 17 stepper motor’s shaft via two M3 nuts and two M3 × 16 mm hex bolts. The gear is designed to be self-centering on the motor shaft.
**Large Gear (**Fig. 2H**):** The large gear mounts onto the leadscrew of the system and meshes with the small gear mounted to the NEMA 17 stepper motor. The large gear is fixed to the leadscrew using two counter-tightened M6 nuts, one on either side of the gear (see Fig. 3I). The large gear should be mounted last during assembly.
**Syringe Guide (**Fig. 2I**):** PrintrBot-specific LVE mount connecting the LVE to the Y-axis of the PrintrBot Simple metal. Two slots for M3 nuts and holes for (1) screwing the LVE to the Syringe Guide, (2) screwing the syringe guide to the PrintrBot, and (3) screwing the Syringe Collar into the syringe guide using M3 × 10 mm hex bolts.
**Syringe Collar (**Fig. 2J**):** Collar designed to keep the syringe in place. It screws into the Syringe Guide after the 60 mL syringe is inserted into LVE for printing (Fig. 4B).
**Needle Adapter (**Fig. 5C**):** Adapter designed specifically for mounting the needle to the PrintrBot. There are two slots for M3 nuts, and two corresponding holes into which the Needle Collar is screwed using M3 × 10 mm bolts. Likewise, there are three holes that match to those pre-existing holes on the PrintrBot Simple Metal and with which the Needle Adapter is mounted onto the printer.
**Needle Collar (**Fig. 5D**):** Functions to keep the needle in place; screws into the needle adapter using M3 × 6 mm bolts.

## 4. Bill of materials

**Table:** 

Designator	Component	Number of Units	Cost per unit [USD]	Total Cost [USD]	Source of Materials	Material Type
Double Shielded Ball Bearings	608-ZZ	2	0.70	1.40	Amazon	Steel
M8 × 2.4 mm Steel Hex Nuts	90592A022	4	0.05	0.19	McMaster-Carr	Steel
M8 Brass Hex Nuts	MN2580000BR0000	2	0.50	1.00	Fastenal	Brass
M8 – 1.25 × 1 m, Threaded Rod, Stainless Steel, 304	21YP40	1	10.00	10.00	Grainger	Steel
M3 × 10 mm Socket Head Screws (i.e. Hex Bolts)	91290A115	10	0.07	0.70	McMaster-Carr	Steel
M3 × 16 mm Socket Head Screws (i.e. Hex Bolts)	91290A120	4	0.08	0.32	McMaster-Carr	Steel
M3 × 8 mm Socket Head Screws (i.e. Hex Bolts)	91290A113	2	0.07	0.14	McMaster-Carr	Steel
M3 × 45 mm Partially-Threaded Socket Head Screws (i.e. Hex Bolts)	91290A079	2	0.14	0.28	McMaster-Carr	Steel
M3 Hex Nuts	90592A085	16	0.01	0.14	McMaster-Carr	Steel
60 mL Luer-Lock BD Syringe	TM82175	1	0.77	0.77	Amazon	Polypropylene
Standard Polyurethane Tubing, 1/8″ OD, 1/16″ ID	4HL93	1	16.00	16.00	Grainger	Polyurethane
18-Gauge, 4″ long blunt-tip needle with Female Luer Lock Connection	6710A44	1	4.01	4.01	McMaster-Carr	Stainless Steel
Male Luer to 1/16″ Tubing Barb	EW-45,518-00	1	0.48	0.48	Cole-Parmer	Polypropylene
Epoxy	554,869,652	1	6.95	6.95	Walmart	Steel Reinforced Epoxy
17-Gauge Luer-Lock Needle	JG17-1.0X	1	0.18	0.18	Jensen Global	Stainless Steel
26-Gauge Luer-Lock Needle	75165A555	1	0.24	0.24	McMaster-Carr	Stainless Steel
Loctite 0.2 fl. oz. Threadlocker Blue 242	209728	1	6.47	6.47	Home Depot	Glue/Epoxy

## 5. Build instructions

A majority of the LVE components are 3D printed. The remaining hardware can be acquired online from common hardware suppliers such as McMaster-Carr and Grainger (see *Bill of Materials*). Complete step-by-step build instructions are also provided in supplementary video 1.

Hand tools required for assembly include:

- M3 Allen wrench
- M8 wrench
- Slip-Joint Pliers

### 5.1. Build instructions for the mounted LVE (Fig. 3)

1 3D print all the STL files (one of each) listed in the *Design Files Summary*.
  - Use of PLA, high infill (70%), and at least two perimeters are recommended when printing the Core to maximize strength under compression.
  - None of the parts require support material during printing *except* the syringe plunger (Syringe Plunger.STL), which should be printed in an orientation such that the hollow inner cavity faces the build plate during printing (See Fig. 3A for an image of the plunger orientation during printing).
2 Cut the threaded rod to size: 300 mm.
3 Build the Nut Shuttle assembly by inserting two M8 brass nuts into the Nut Shuttle (Nut Shuttle.STL) and screwing the Nut Shuttle Plate (Nut Shuttle Plate.STL) into place (see Fig. 3B). Place M3 hex nuts (three) into the hex-nut-shaped indentations in the Nut Shuttle Plate, which should face outward.
  - If needed, insert a washer in-between the two brass nuts to reduce friction in the final Nut Shuttle assembly.
  - Depending on the fidelity of the 3D printed parts (which can vary among desktop printers), the brass nuts may be difficult to insert into the Nut Shuttle since it was designed to be tight-fitting to minimize slack in the system. In this case, hollowing out the nut hole using hand tools and sand paper, or re-printing the nut shuttle with different printer settings, can solve this problem.
  - Do not over-tighten the three bolts attaching the Nut Shuttle Plate to the Nut Shuttle; the plate should remain relatively flat. Overtightening the bolts will cause the plate to bend and, in the case of PLA, creep over time. Likewise, the bolts should be approximately equally-tightened.
4 Once assembled, screw the Nut Shuttle onto the threaded rod a distance approximately 75 mm. The assembled nut shuttle should be oriented on the threaded rod such that the Nut Shuttle Plate was screwed onto the rod first and the bottom of the Nut Shuttle (where the Syringe Plunger will later slide into place) faces the closest end of the rod. Note that this end of the rod will sit in the guide post of the Core once the assembly is complete.
5 Screw one M8 steel nut and one 608-zz bearing onto the end of the leadscrew opposite to where the Nut Shuttle currently resides. Screw the M8 steel nut on first, followed by the 608-zz bearing. Space them along the threaded rod such that the nut and bearing reside to the left of the bearing housing of the LVE core, noting that the opposite end of the threaded rod will sit in the guide post. Refer to Fig. 3C for an explanatory image of this spacing.
6 Snap the bearing into place in the bearing housing by pulling the threaded rod to the right, then screw the nut against the bearing, making sure that the threaded rod remains sitting in the guide post. Do not fully-tighten yet.
7 Screw the other M8 nut and 608-zz bearing onto the leadscrew and into the bearing housing on the LVE core. Using Loctite, counter-tighten the M8 steel nuts against the bearings, first by hand, and then by using the slip-joint pliers to hold the left nut steady while using the M8 wrench to tighten the right nut.
  - Prior to tightening the nuts, check to make sure that the threaded rod sits in, and no further than, the guide post (See Fig. 3D).
  - When applying Loctite to the threaded rod, do not get any Loctite on the bearings.
  - After tightening the nuts, spin the threaded rod several times to make sure the nuts have not been over-tightened and are not inhibiting the rotation of the rod. The rod should spin freely. If you feel significant friction, loosen the nuts in quarter-turn increments as needed.
  - Allow the Loctite to cure for the required amount of time before printing with the LVE.
  - Note that nylon lock nuts are an alternative to steel M8 nuts with Loctite, and they will not leave residue behind upon removal.
8 Mount the small gear (Small Gear.STL) onto the NEMA 17 stepper motor using two M3 nuts and two M3 × 16 mm hex bolts. Tighten as much as possible to prevent loosening of the small gear from the motor shaft during printing (see Fig. 3E).
9 Mount the NEMA 17 stepper motor (with the small gear attached) to the LVE Core using three M3 × 16 mm screws.
10 Mount the LVE to the printer.
  - In connecting the LVE to a PrintrBot Simple Metal, we designed an additional part, the Syringe Guide (Syringe Guide.STL). It contains two slots for M3 nuts in the Syringe Mount (See Fig. 3F) where the Syringe Collar (Syringe Collar.STL) screws into place. The Syringe Guide also screws into the LVE using two M3 × 10 mm hex bolts and two M3 nuts (Fig. 3G).
  - Mounting the LVE core to the PrintrBot required the use of two M3 × 45 mm partially-threaded bolts which threaded through the two holes found on either side of the Core’s bearing casing, and also two M3 screws that came with the PrintrBot. See Fig. 3H for an image of the fully-mounted LVE.
  - The LVE is compatible with a variety of printers, though the mounting scheme for each printer may be different and require uniquely-designed adapters. Mounting onto a MakerBot replicator, for example, could be as simple as strapping the core to the frame with Velcro. Designing a simple and 3D printable mounting adapter may be required; the CAD files of all LVE components are shared for this reason.
11 With the LVE core mounted onto the printer, the last step is to attach the large gear (Large Gear.STL) to the threaded rod. Do so by first threading an M8 steel nut onto the rod, followed by the large gear, and then by another steel nut. Mesh teeth of the large gear with the small gear and tighten the Large Gear into place by counter-tightening the two steel nuts using the M8 wrench and pliers. See Fig. 3I. Unscrewing one of the bolts attaching the NEMA 17 Stepper Motor to the LVE Core, enabling the motor to rotate about the remaining screw keeping it attached to the LVE Core, will make the gear-meshing process easier. Once the gears have been meshed, re-insert and tighten the screw.

### 5.2. Syringe preparation and insertion instructions (Fig. 4)

12 Remove the stock plunger from the 60 mL BD syringe and remove the neoprene cap. Put the cap onto the 3D-printed plunger. See Fig. 4A.
13 Load the 60 mL syringe with your fluid material of choice and install the syringe into the LVE, inserting the syringe flange into the designated slot in the LVE and screwing the Syringe Collar into place to keep the syringe from disengaging from the printer during use. See Fig. 4B.
  - Purge the syringe (and Bowden system) of all air bubbles. Air bubbles compress within the system during operation and cause the extrusion of incorrect amounts of materials. We recommend degassing your fluid material prior to loading it into the syringe.
  - Prior to filling the syringe, one can run a weak dish soap solution through the syringe and Bowden system to make the removal of bubbles from the system easier during material loading.

### 5.3. Needle Preparation and attachment instructions Fig. 6

**Fig. 6:**
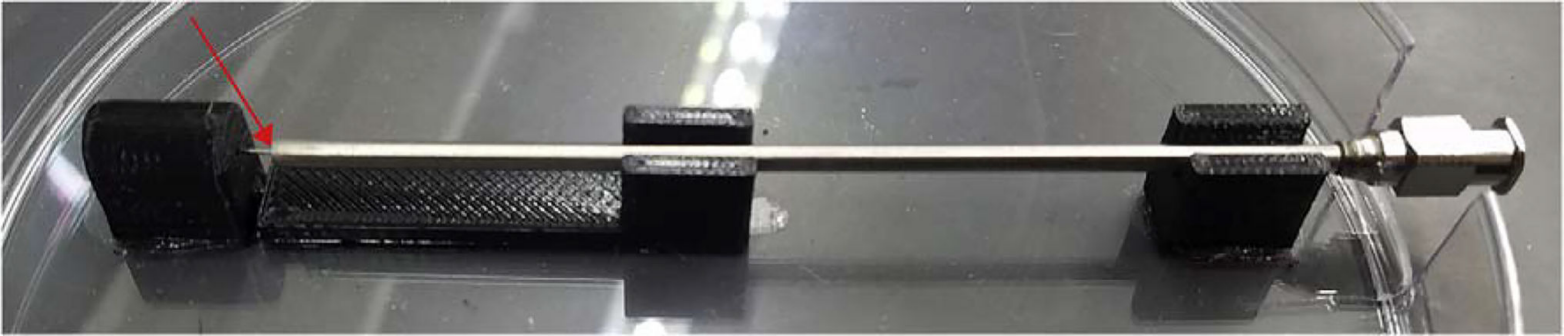
Jig for Application of Epoxy to Needles. The three needle jigs provided in the “Design Files Summary” above are here printed in black PLA and used to align the 18-gauge and 250 µm needles for epoxying. The 100 mm-long, 18-gauge needle sits in the right-most and center needle-jig (“18-Gauge Needle Jig_short.STL” and “18-Gauge Needle Jig_extended.STL”). The metal tip of the 250 µm needle, after removal from its plastic luer, is held by the left-most jig in this image. The red arrow indicates where the epoxy is applied to bind the two needles together. In this instance, the jigs were aligned and affixed to a petri-dish using silicone vacuum grease. A rectangular cut was made in the petri dish to make room for the 18-gauge needle.

14 To make the extended needle, gently remove the plastic luer-adapter from the 250 µm needle by heating up the needle in hot water and then pulling the pieces apart. Using the 3D printed needle-jigs, which act to center the 250 µm needle inside the tip of the 18-gauge needle and keep them both in place during epoxy curing, slide the 250 µm needle into the 18-gauge needle approximately 2 mm and then epoxy it in place, making sure not to clog the entrance to the 250 µm needle. See Fig. 6 for an image of this set-up. Allow the epoxy to cure for the required amount of time.
15 To assemble the Bowden portion of the LVE, first cut the polyurethane tubing. Required tubing length will vary with printer; here, we cut approximately 22 mm. In one end of the tubing, insert the male luer-to-tubing barb and screw the adapter into the 18-gauge needle. Force the tip of the 17-gauge needle into the other end of the tubing and screw the needle into the 60 mL syringe containing your material.
16 To attach the needle to the printer, remove the printer’s stock plastic hot-end and affix the epoxied needle to the extruder carriage assembly. For the PrintrBot Simple Metal, we designed a simple bracket into which the needle can be inserted (Needle Adapter.STL) and a screw-in collar (Needle Collar.STL) to hold the needle in place using M3 nuts and M3 × 8 mm hex bolts. See Fig. 5E.

An instructional, step-by-step video showing assembly and mounting of the LVE to the PrintrBot Simple Metal can be found in Supplementary Materials. Refer to Fig. 1 for an image of all the components fully assembled and installed.

## 6. Operation instructions

The 3D printer and open source software being used with the LVE will vary according to user preference, however the main operation instructions are as follows:

- Load a syringe of material into the LVE and connect its tubing and needle.
- Turn on the 3D printer
- Generate the G-code of the object to be printed using open source software such as Slic3r, Cura, or Matter Control
- Manually place the needle at the center of the area where the bottom layer of the object is to begin printing
- Load and execute the G-code
- If FRESH printing:
  ◦ If printing alginate via the FRESH method, print into a container of FRESH support material
  ◦ Release printed object after printing has completed by submerging the container of solution in a warm, 42 °C 0.1% w/v CaCl_2_ solution.

The LVE was designed for use with open source software and with NEMA 17 stepper motors. Thus, it can be operated via the same software that operated the printer prior to modification; a user need only make a new settings profile within their chosen software and separately alter the firmware value for “extruder steps/mm” on the printer. In this case, we use Slic3r to generate G-code and Pronterface as the host by which to interface with the PrintrBot and interact with the firmware. Essentially, the LVE operates very similarly to a common thermoplastic FDM printer in that it runs on the same G-code file as would be generated for a plastic printer.

A key change that must be made in the software during G-code generation to include the following commands:

**Table:** 

M302	; Enable cold-extrusion
G92 X73.5 Y70.5 Z0.00 E0.00	; Tell the printer that the nozzle is homed and at a height of zero in Z to enable printing to begin at the manually-selected point. The X and Y values used correspond to one half the length of the X and Y build dimensions of the printer platform; the values used here are for a PrintrBot Simple Metal.
T0	; Set the nozzle temperature to zero.

Make sure to disable homing on your printer to prevent the needle from moving to an undesired location at the beginning of printing.

The firmware in your 3D printer must also be altered to include the following steps per mm value, calculated according to the number of microsteps required to advance the 60 mL plunger by 1 mm in either direction: 9255.38 steps/mm.

When printing into a container of FRESH support material, it is recommended that the container be affixed to the print bed using a material like silicone vacuum grease. This prevents the containers from sliding around during printing.

It is recommended, but not always necessary, to manually prime the needle before printing to ensure that upon beginning a print, the fluid in the Bowden system begins to be extruded.

Air bubbles are a major concern during operation because they contract under compression and cause an incorrect amount of extrusion during printing. Attempt to purge all air bubbles from the system prior to printing.

Needle clogs are a possibility. Be aware of a clogged needle because it can cause back-pressure build-up in the Bowden system and often result in unwanted leakage from the syringe.

## 7. Validation and characterization

**Fig. 7:**
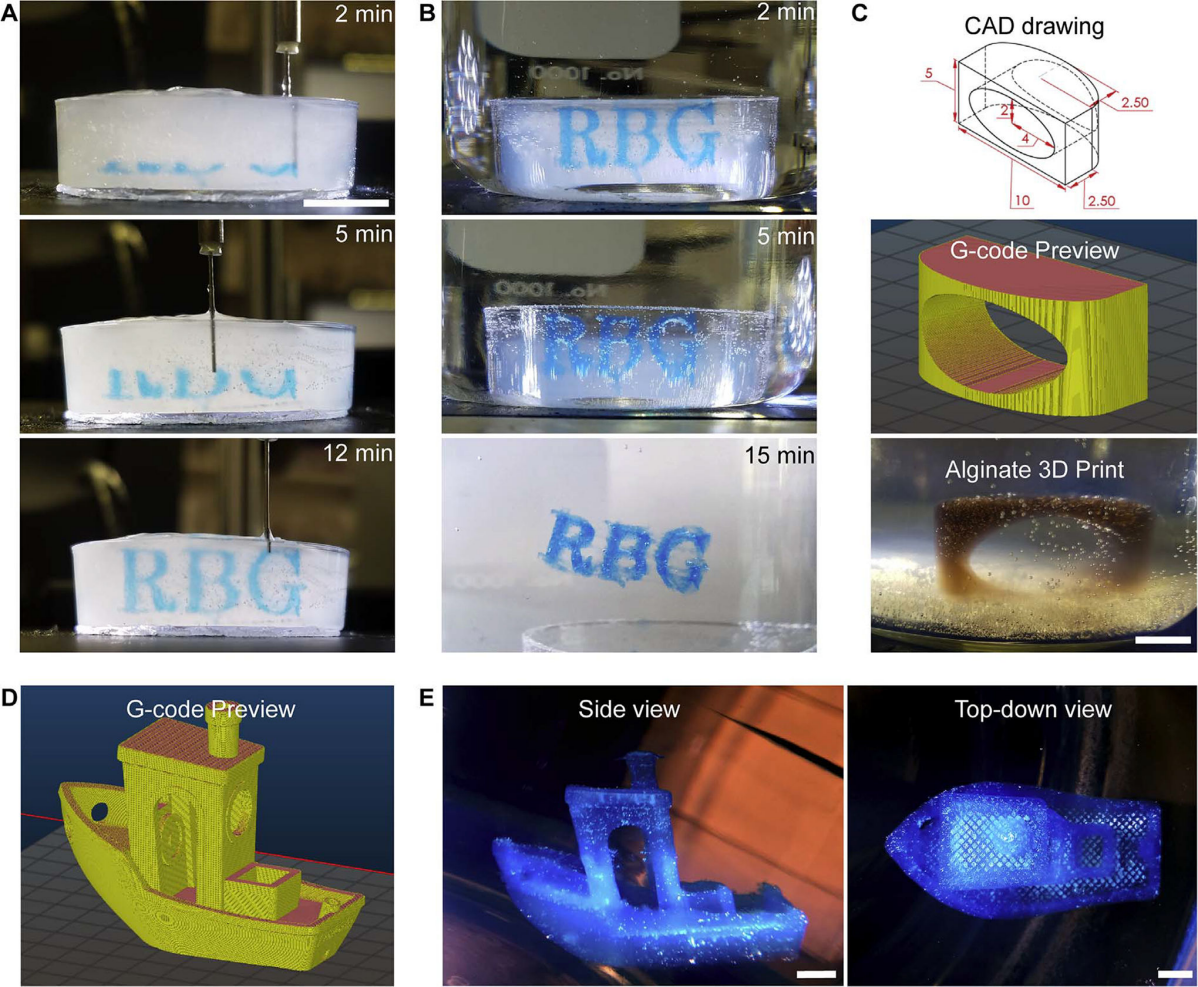
3D printing hydrogels with the LVE. (A) Printing sequence of “RBG” into a petri dish of support material using blue-dyed alginate via the FRESH method [4]. (B) Release of the completed “RBG” from support material after printing has completed by submersion in a 250 mL beaker of warm, 42 °C 1% w/v calcium chloride (CaCl_2_) solution. The final frame in the image series shows the fully-released letters floating in the beaker. (C) 3D drawing (with dimensions in mm) of an EWC print, pathing in Slic3r for a 4× scaled version, and completed and released print of an EWC. (D) 1.35×-scaled 3D Benchy G-code preview in Slic3r. (E) Top-down of the completed and released 3D Benchy printed out of Alcian blue-dyed alginate. All scale bars are 1 cm.

The LVE was used to 3D print a series of calibration prints to validate its accuracy and precision as a Bowden syringe pump extrusion system. As shown in Fig. 7, the letters “RBG” (Fig. 7A and B), a 4x-scaled Elliptical Window Calibration (EWC) (Fig. 7C), and a 1.35×-scaled 3D Benchy (Fig. 7D and E) were printed using alginate via the FRESH method developed by Hinton et al. using the LVE [4]. The printed objects, upon release from the support bath, appeared as intended and corresponded to the G-code pathing previews generated in the open source slicing software, Slic3r. There is always a finite lag between starting and stopping extrusion in a syringe based system, however, we did not observe noticeable defects or impact on print performance at the macroscopic scale, for example with the EWC and 3D Benchy. It is of course possible that this lag may affect print quality at the microscopic scale, but this was not analyzed because the point of the LVE is not to achieve maximum quality, rather it is to print with large volumes.

The EWC is a calibration object used to test a 3D printer’s ability to print steep overhangs and large horizontal holes. Initially 10 mm in length, the EWC was scaled 400% to 40 mm and successfully printed out of red-dyed alginate solution using the LVE, as shown in Fig. 7C. The print was measured to be 39.69 mm in length upon release from the support bath, constituting a percent error of only 0.78% in the X-direction.

The 3D Benchy is a standard benchmarking test within the open source desktop-printing community [16]. It is available for download on the popular file-sharing website Thingiverse (http://www.thingiverse.com) and contains a variety of features that are geometrically challenging to print, such as a large overhanging curved surface (the hull of the ship), small surface details (lettering on the stern of the ship), cylindrical shapes (the chimney), several flat overhangs (the roof and the tops of rectangular windows), low-sloping surfaces, and circular horizontal holes (the windows). We 3D printed the 3D Benchy using 4% w/v Alcian Blue-dyed alginate, 25% infill, and 0.12 mm layer thickness at 35 mm/s. The print took approximately six hours total and was released from the support bath and photographed afterwards. As can be seen in Fig. 7E, the LVE successfully printed the overhangs, windows, low-sloping surfaces, and cylindrical shapes – the features are clearly recognizable and look as expected. The fine-feature lettering on the stern of the ship are unable to be discerned, however this is unsurprising given that the plate containing the lettering is designed to be only 0.135 mm thick and 2.7 mm tall, while the extrusion width of the needle is 0.25 mm (its internal diameter) and the layer thickness is 0.12 mm. The successful printing of the 3D Benchy demonstrates the performance of the LVE as a fluid materials extrusion device. The LVE has here been demonstrated to function as a reliable and inexpensive open source modification for Cartesian desktop printers.

## Supplementary Material

1

2

3
